# Malignant Transformation of Mature Cystic Teratoma: A Case Report and Literature Review

**DOI:** 10.7759/cureus.96643

**Published:** 2025-11-12

**Authors:** Yusuke Nabe, Hiroshi Mizuuchi, Masaaki Inoue, Junichi Yoshida

**Affiliations:** 1 Department of Chest Surgery, Shimonoseki City Hospital, Yamaguchi, JPN

**Keywords:** bep therapy, gctsm, mature teratoma, mediastinal somatic tumor, multimodal treatment

## Abstract

Malignant transformation of mature teratomas is rare, occurring in 1%-3% of cases, and no standard treatment has been established. According to the latest World Health Organization (WHO) histological classification, germ cell tumors with a somatic malignant component (sarcoma, carcinoma, or both) are defined as germ cell tumors with somatic malignancy (GCTSM) and have a poor prognosis. Herein, we report a rare case of a mediastinal mature cystic teratoma that showed malignant transformation. The patient achieved complete remission with multimodal treatment. A 74-year-old female non-smoker presented with a main complaint of right-sided chest pain. Twelve years ago, when she visited a local doctor complaining of a cold, an abnormal shadow was detected on chest computed tomography (CT), but she did not seek treatment at that time. The chest radiography performed now revealed increasing right pleural effusion, and the chest CT suggested a mediastinal tumor; therefore, the patient was referred to our department. Chest CT revealed a large mass shadow with calcification in the anterior mediastinum. Although the anterior mediastinal tumor was suspected to have infiltrated the right middle and lower lobes, parietal pleura, and pericardium, complete resection was possible, and surgery was performed for both diagnosis and treatment. Intraoperative findings showed that the tumor had infiltrated the inferior pulmonary vein, resulting in a gross incomplete resection. The pathological diagnosis was mature teratoma with a primary mediastinal somatic malignant tumor. Three courses of etoposide, cisplatin, and bleomycin (BEP) therapy were administered as primary chemotherapy. Three years have passed since the surgery, and the patient has remained recurrence-free. As in this case, BEP therapy is effective in some cases, and further accumulation of such cases will be necessary in the future.

## Introduction

Mature teratomas are characterized by a haphazard distribution of mature somatic cells, with skin and skin appendages forming the cystic lining, as well as bronchial mucosa and glands, gastrointestinal mucosa, nervous and mature brain tissue, smooth muscle, adipose tissue, and pancreatic tissue being present in 60%-80% of cases [1].

Malignant transformation of mature teratomas is rare, occurring in 1%-3% of cases, and transformations to sarcomas, adenocarcinomas, squamous cell carcinomas, and carcinoid tumors have been reported [2]. The mechanism of malignant transformation is of two types: (i) chemotherapy or radiation therapy induces malignant transformation in a part of a germ cell tumor (more common in younger people) and (ii) part of a teratoma is thought to have transformed into a malignant tumor over the natural course of the disease (more common in older people) [3].

Among malignant tumors with sarcomatous components, rhabdomyosarcomas are most common, followed by angiosarcomas, leiomyosarcomas, liposarcomas, and undifferentiated sarcomas [4]. Given that the malignant transformation of mature cystic teratomas is rare, there have been no large-scale prospective trials, and no standard treatment has been established.

According to the latest World Health Organization (WHO) histological classification, germ cell tumors with somatic malignant components (sarcoma, carcinoma, or both) are defined as germ cell tumors with somatic malignancies (GCTSMs), and GCTSMs have a poor prognosis, with a median patient survival of approximately nine months; rarely, patients survive more than three years [1].

Herein, we report a rare case of a mediastinal mature cystic teratoma that showed malignant transformation, and complete remission was achieved with 3-kur etoposide, cisplatin, and bleomycin (BEP) therapy.

## Case presentation

A 74-year-old female non-smoker presented with a chief complaint of right-sided chest pain. Previously, when she visited a local doctor for a cold, an abnormal shadow was detected on a chest CT, but she did not request treatment. Twelve years later, when she visited a local doctor with right-sided chest pain, chest radiography revealed right pleural effusion, and the patient was treated with disopyramide. The right pleural effusion tended to increase, and the patient was referred to our hospital's cardiology department. A chest CT raised a suspicion of a mediastinal tumor, and the patient was referred to our department. Her medical history included basal cell carcinoma of the face, high blood pressure, and hypercholesterolemia. Table 1 presents the blood test results.

**Table 1:** Blood test results

Parameters	Results	Unit	Normal Range
Alpha-fetoprotein	3.4	ng/mL	0.4–10
Carcinoembryonic antigen	2.4	ng/mL	0–5
Cancer antigen 125	96.8	U/mL	35 or less
Squamous cell carcinoma antigen	0.9	ng/mL	1.5 or less
Cytokeratin-19 fragment	2.4	ng/mL	0–3.5
Neuron-specific enolase	29.7	ng/mL	0–16.3
Pro-gastrin-releasing peptide	41.3	pg/mL	0–81
Anti-acetylcholine receptor antibody	<0.3	nmol/L	0–0.3

**Figure 1:**
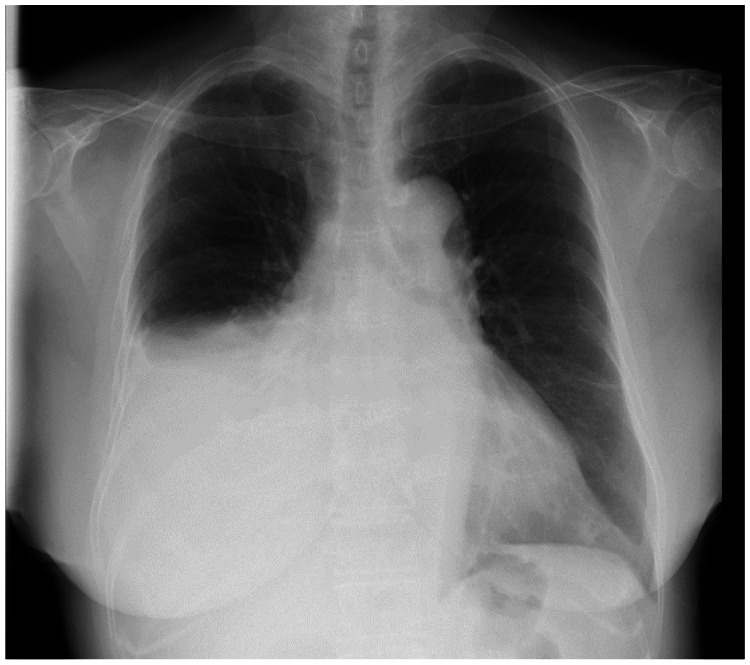
Initial chest radiography findings Right pleural effusion can be observed.

**Figure 2:**
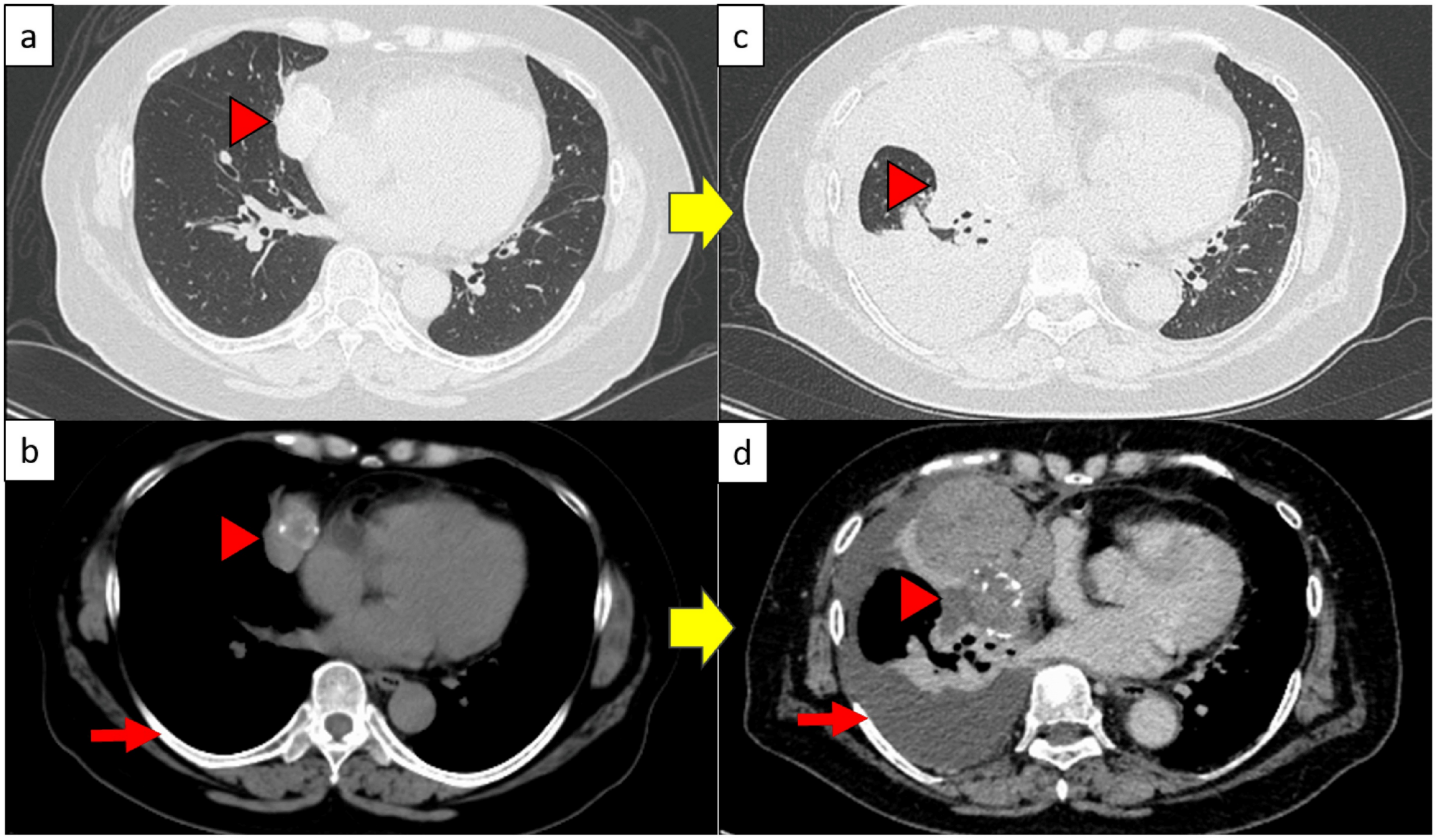
Chest computed tomography (CT) results The image findings in panels (a) and (b) comprise CT images taken 12 years earlier, and those in (c) and (d) are initial chest CT results. (a) A mass shadow was observed in the anterior mediastinum. No nodular shadows were observed due to pulmonary metastasis. ▶: Mass (b) The mass shadow in the anterior mediastinum from (a) measured 45 × 41 × 28 mm and was accompanied by calcification. ➡: No pleural effusion was observed. ▶: Mass (c) Tumor growth- and pleural effusion-related compressive atelectasis was observed. ▶: Compressive atelectasis (d) The tumor reached the size of 95 × 82 × 80 mm and right pleural effusion was noted. ➡: Pleural effusion was observed. ▶: Mass

Figure 1 displays the chest radiography findings from the initial visit. Chest CT revealed a large mass with calcification in the anterior mediastinum (Figure 2).

Cardiac function was normal. Pulmonary function tests revealed the following: forced vital capacity, 1.03 L (51%); forced expiratory volume in 1 s, 0.92 L (58.2%); FEV1.0 percent predicted, 89.32%; and diffusing capacity of the lung for carbon monoxide, 9.87 mL/min/mmHg (67.2%). Restrictive ventilatory disorder was observed.

**Figure 3:**
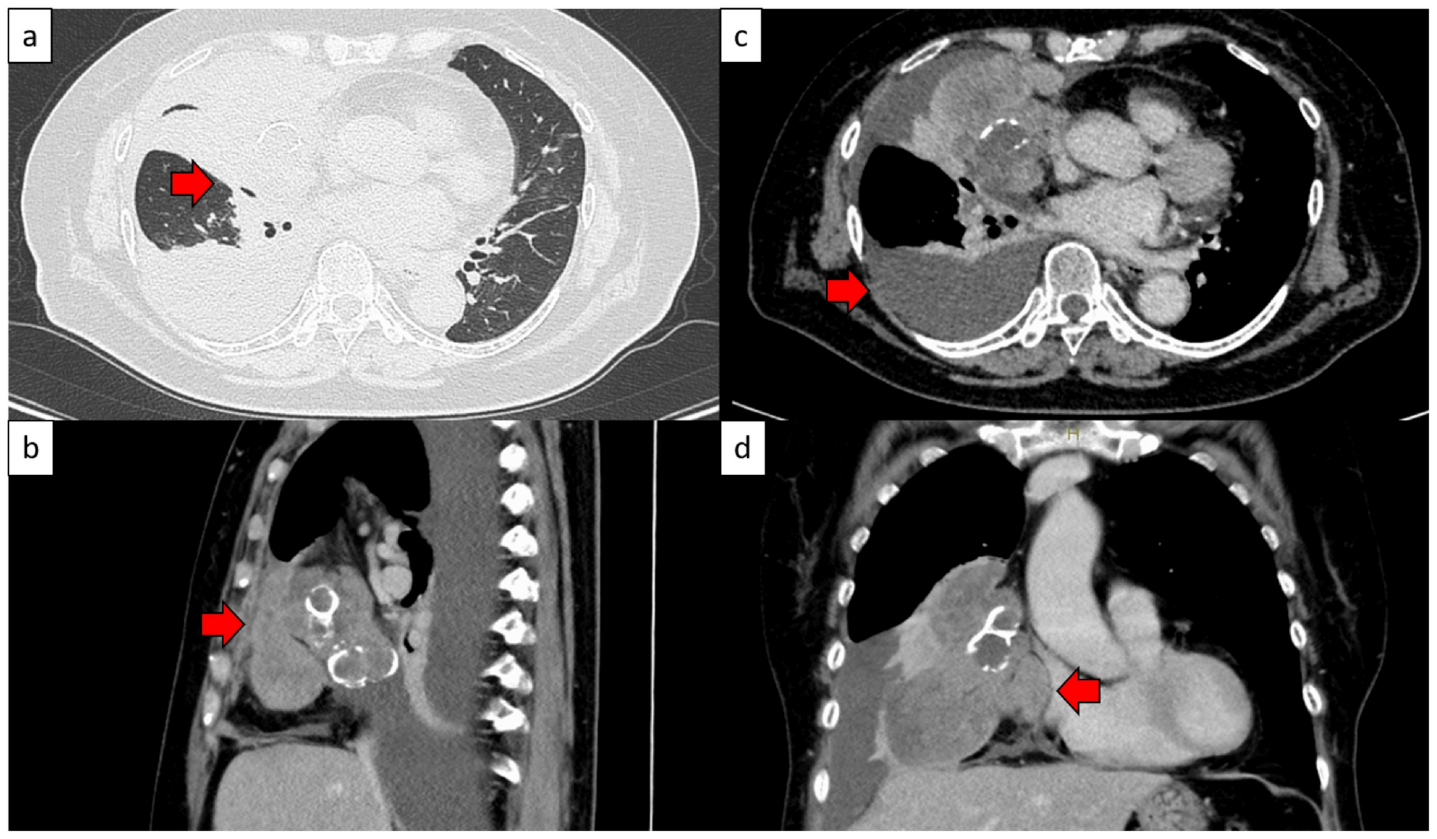
Preoperative chest computed tomography (CT) results (a) Infiltration into the right middle and lower lobes was suspected. ➡: Suspected infiltration into the right middle and lower lobes (b) Although potential right pleural infiltration was observed, apparent right chest wall infiltration was not. ➡: Right pleural infiltration (c) Right pleural effusion was observed. ➡: Right pleural effusion (d) Although suspected epicardial infiltration was observed, intrapericardial infiltration was not. ➡: Suspected epicardial infiltration

The anterior mediastinal tumor was suspected to have infiltrated the right middle and lower lobes, parietal pleura, and pericardium (Figure 3). We determined that complete resection was possible and decided to perform surgery for both diagnosis and treatment.

Under general anesthesia and with the patient in the left lateral position, a thoracotomy was performed via a posterolateral incision to remove the mediastinal tumor.

**Figure 4:**
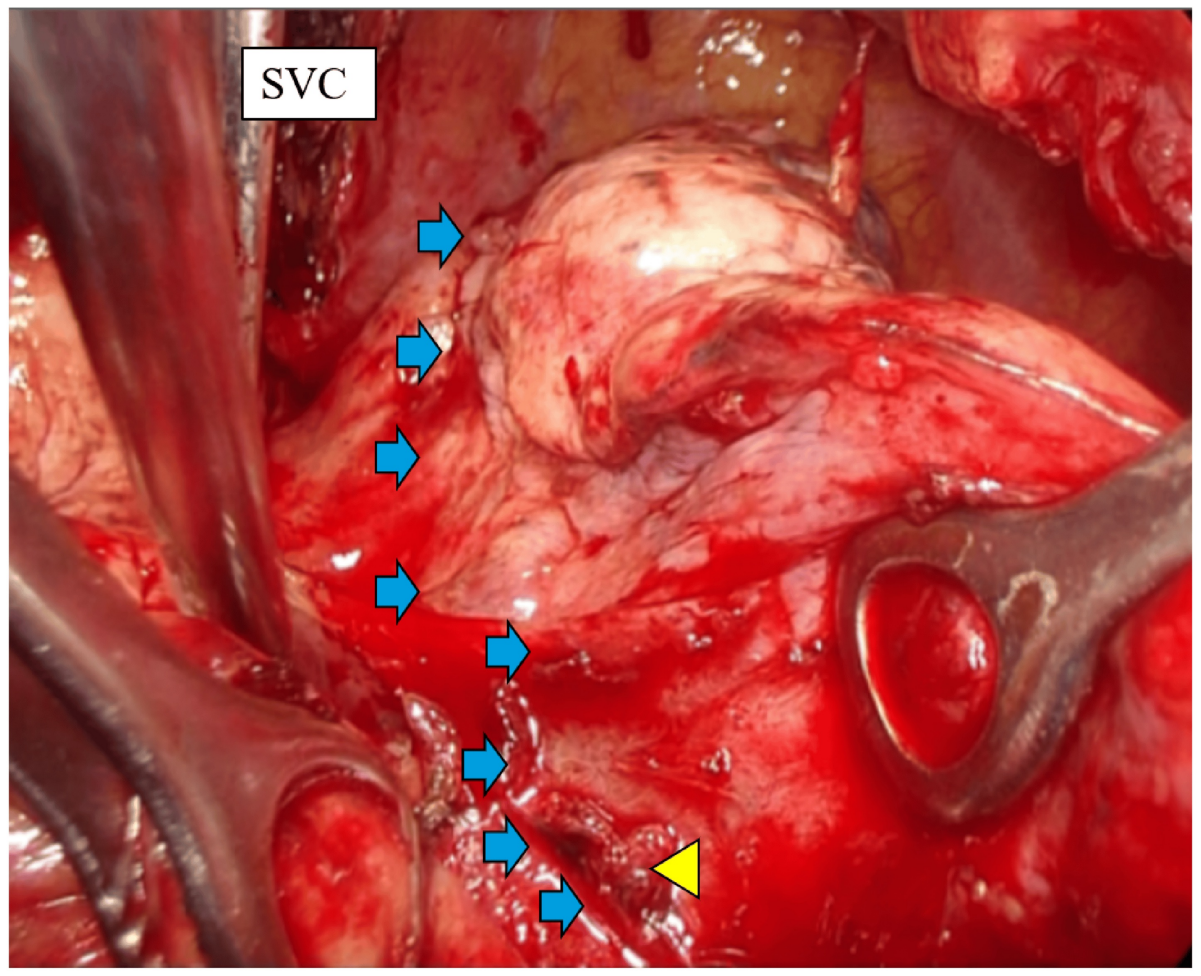
Surgical findings (right intrathoracic cavity) Surgical findings showed infiltration around the inferior pulmonary vein, making complete resection difficult. ➡: Tumor border. ▶: Invasion of the right inferior pulmonary vein SVC: superior vena cava

Intraoperative findings revealed tumor infiltration in the middle and lower lobes. Complete resection was deemed difficult due to infiltration of the inferior pulmonary vein. The surgery was completed with an incomplete macroscopic resection, leaving a portion of the tumor around the inferior pulmonary vein (Figure 4). Blood loss was 2300 mL, and the operative time was 5 hours 26 minutes.

**Figure 5:**
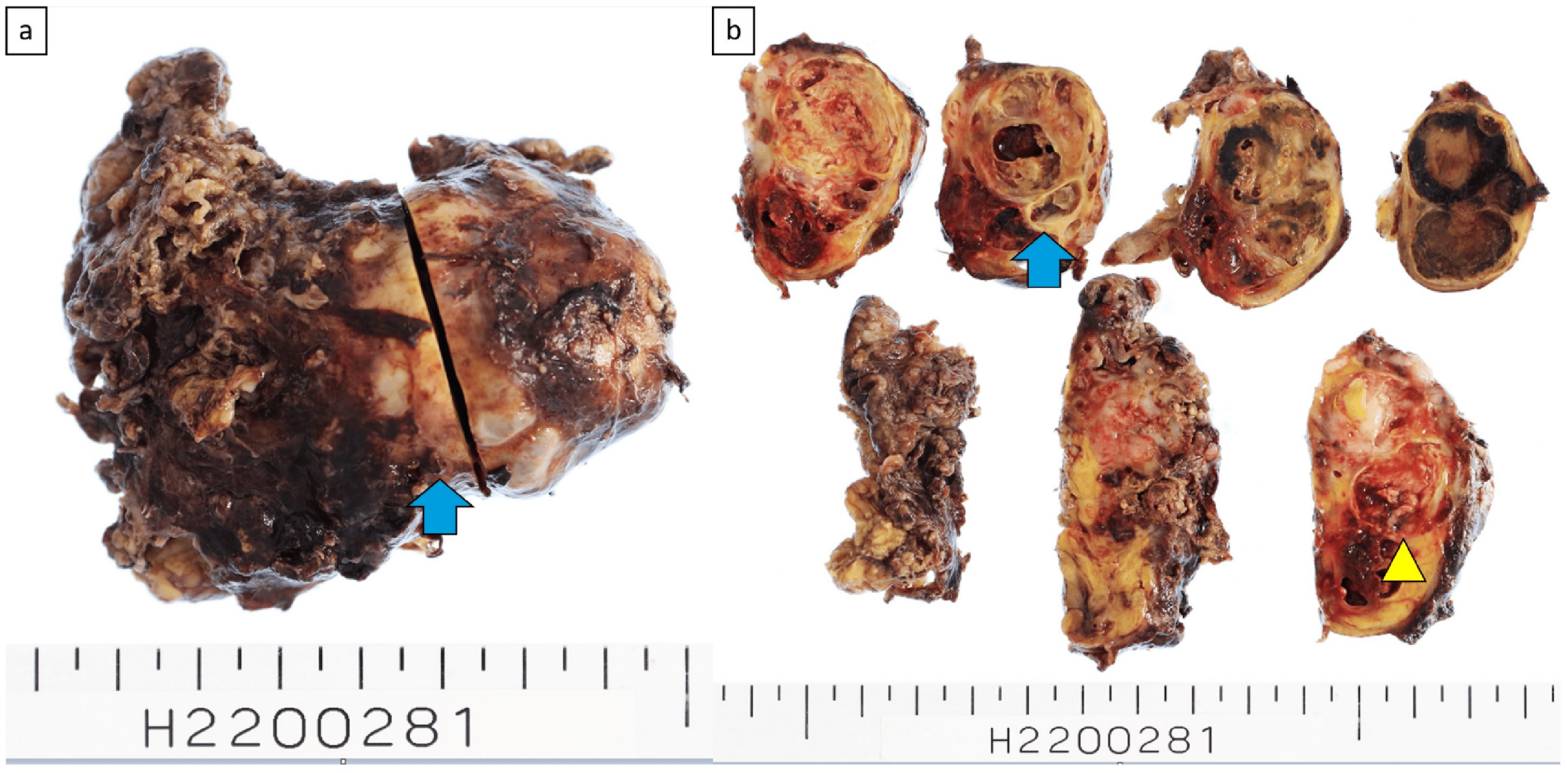
Specimen photograph (a) A capsule with calcification was observed on the tumor surface. ➡: Capsule with calcification (b) The cut surface of the tumor revealed multicystic and soft fleshy areas with hemorrhagic necrosis. ➡: Multicystic areas. ▶: Soft fleshy areas with hemorrhagic necrosis

**Figure 6:**
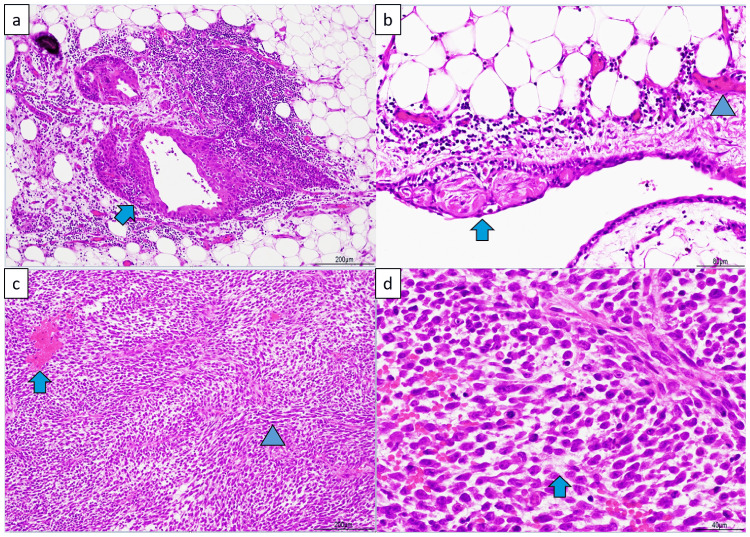
Pathological findings (H&E staining) (a) The cysts contained necrotic material; although many had lost their epithelium, some were lined with stratified squamous epithelium. ➡: Stratified squamous epithelium (b) Fatty tissue and blood vessels were found in the surrounding area. ➡: Stratified squamous epithelium. ▶: Blood vessels (c) From the cystic to fleshy areas, spindle-shaped or bare-nucleated atypical cells proliferate extremely densely with noticeable hemorrhagic necrosis. The nuclei were rich in fine chromatin and contained one or two spindle-shaped nucleoli with sharp ends. The pleomorphism was not strong. Many mitotic figures were observed (15 per 10 HPF). ➡: Hemorrhagic necrosis. ▶: Dense proliferation of spindle-shaped or bare-nuclear atypical cells (d) A flowing arrangement and myxoid changes in the interstitial tissue were observed. ➡: Interstitial myxoid changes H&E: hematoxylin and eosin

**Figure 7:**
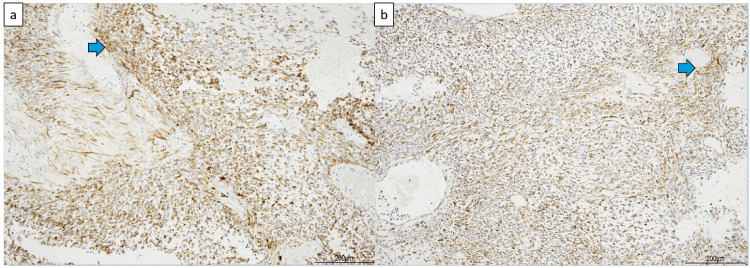
Immunostaining results (a) Immunostaining for the epithelial marker CAM5.2. ➡ Positive cells (b) Immunostaining for the mesenchymal marker vimentin. ➡ Positive cells

The tumor had a calcified capsule, and the cut surface revealed multicystic and soft fleshy areas with hemorrhagic necrosis (Figure 5). Figure 6 displays the pathological findings. Pathology showed that the cystic areas contained necrotic material and that most cysts had lost their epithelium, but some were lined with stratified squamous epithelium. Fatty tissue and blood vessels were visible in the surrounding area. From these cystic to fleshy areas, spindle-shaped or bare-nucleated atypical cells proliferated extremely densely, with prominent hemorrhagic necrosis. The nuclei were rich in fine chromatin and contained one or two nucleoli, with the spindle-shaped cells having sharp ends. Pleomorphism was not strong. Mitotic figures were numerous, with 15 per 10 HPF. A flowing arrangement and myxoid changes in the interstitium were observed. Immunostaining was positive for the epithelial marker CAM5.2 and the mesenchymal marker vimentin (Figure 7). Tests for desmin, smooth muscle actin, S100, human melanoma black 45, neuron-specific enolase, chromogranin, synaptophysin, CD56, leukocyte common antigen, factor VIII, and glial fibrillary acidic protein were negative. The myoglobin levels were difficult to determine. Additional immunohistochemistry revealed negative results for cytokeratin (AE1/AE3) and positive results for CD99 (Mic 2), but these were not conclusive owing to nonspecific staining. Myoglobin staining using a lower antibody concentration was equivocal. The diagnosis was mature teratoma with a somatic-type malignancy of the mediastinum.

**Figure 8:**
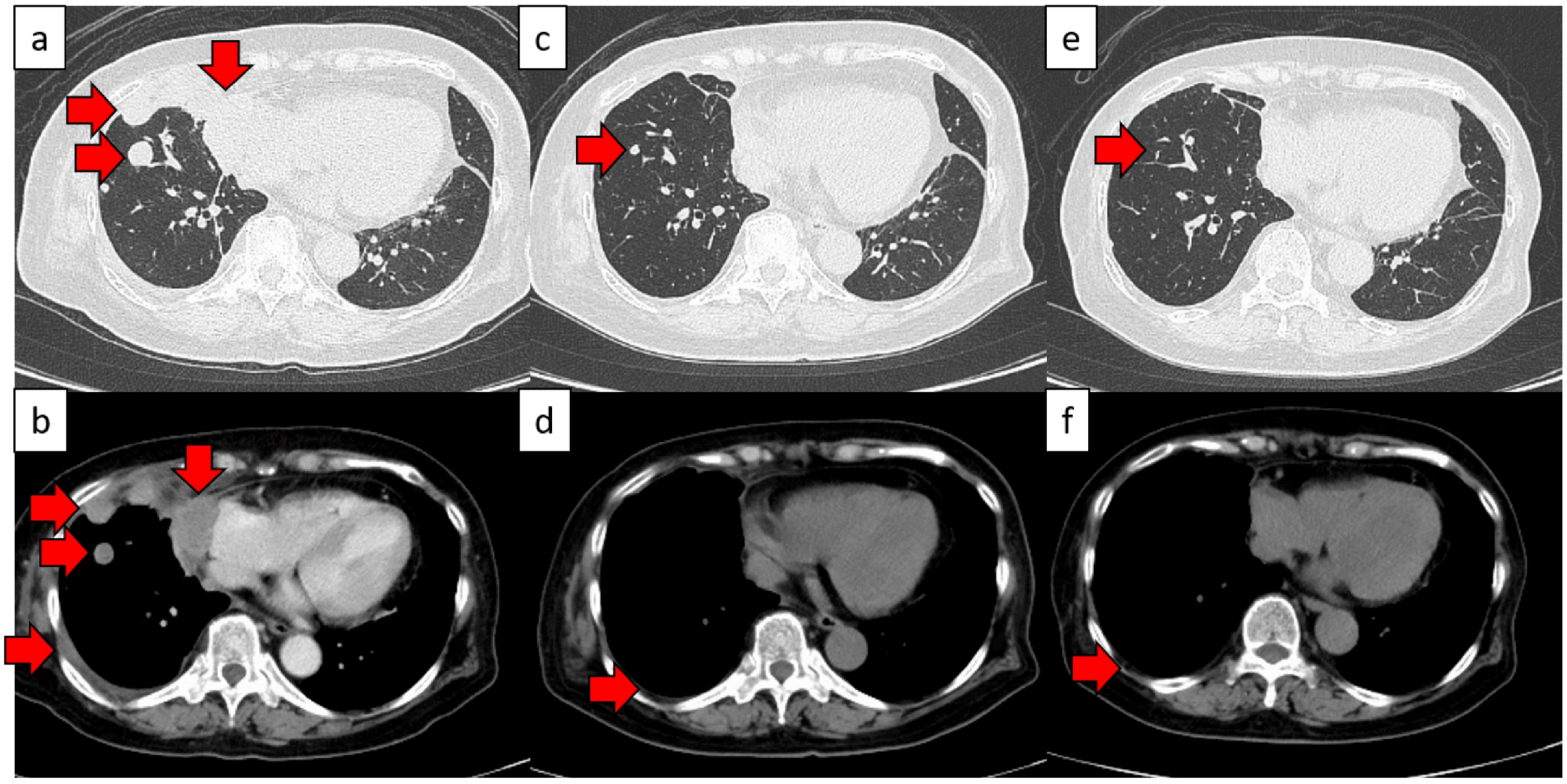
CT results before and after chemotherapeutic treatment (a, b) CT scans before chemotherapy. Lung metastasis and pleural dissemination were observed. ➡ Lung metastasis and pleural dissemination (c, d) CT scans three months after the start of chemotherapy. (c) The metastatic lung tumor and pleural dissemination tended to shrink. ➡ Metastatic lung tumor exhibiting a tendency to shrink. (d) Reduced right pleural effusion was observed. ➡ Reduced right pleural effusion (e, f) CT scans six months after the beginning of chemotherapy. (e) The metastatic lung tumor disappeared, and complete remission was considered to be achieved. ➡ Disappearance of the metastatic lung tumor. (f) The right pleural effusion disappeared. ➡ Disappearance of the right pleural effusion

As first-line chemotherapy, three courses of BEP therapy (etoposide 100 mg/m², cisplatin 20 mg/m², and bleomycin 30 mg/body) were administered. The first course was discontinued after day five, owing to grade 3 fatigue. The second and third courses were reduced by 20% and continued until day three. CT scans performed six months post-chemotherapy initiation showed that the tumor had disappeared, and the patient achieved complete remission (Figure 8).

Three years have passed since the surgery, and the patient has been recurrence-free.

## Discussion

Patients with mature teratomas are often asymptomatic [5]. However, in a small number of patients, the tumor may invade the surrounding mediastinal tissues and undergo malignant transformation; these patients may present with other symptoms such as chest pain, chest tightness, and cough [6]. When a mediastinal germ cell tumor transforms malignantly, the best treatment is complete resection and chemotherapy appropriate for the transformed histological type [7].

Chemotherapy based on somatic histology is recommended, particularly for patients with unresectable malignant transformation [5]. Mature mediastinal cystic teratomas have a wide range of lesions, and severe adhesions and compression between the tumor and surrounding tissue structures can cause severe bleeding during surgery [8]. In this case, strong adhesion around the tumor was noted, making surgery difficult. Ultimately, macroscopic incomplete resection was required owing to tumor infiltration into the inferior pulmonary vein. In this case, sarcomatoid carcinoma or rhabdomyosarcoma with positive epithelial markers on immunohistochemistry were considered as the differential diagnoses; however, as the pattern was atypical and the myoglobin staining results were unclear, the nature of the malignant component was difficult to establish. Therefore, the detailed histological types could not be determined.

Pulmonary sarcomatoid carcinoma (PSC), a heterogeneous tumor, is classified as a non-small cell lung cancer and exhibits features of both epithelial and mesenchymal tissues [9]. It is divided into five subtypes: pleomorphic carcinoma, spindle cell carcinoma, giant cell carcinoma, carcinosarcoma, and pulmonary blastoma [10]. Pathological findings of PSC have been reported as malignant spindle cell tumors on hematoxylin and eosin staining, and as cytokeratin (+) and vimentin (+) on immunohistochemistry [11].

Rhabdomyosarcoma is a rare malignant tumor arising from mesenchymal tissue and is histologically characterized by various stages of rhabdomyoblast differentiation [12].

The WHO classifies rhabdomyosarcoma into different subtypes: embryonal rhabdomyosarcoma, alveolar rhabdomyosarcoma, pleomorphic rhabdomyosarcoma, spindle cell/sclerosing rhabdomyosarcoma, and ectomesenchymoma [13].

Small cell carcinoma was ruled out because chromogranin, synaptophysin, and CD56 tests were negative. Malignant melanoma was ruled out because the S100 protein test was negative. Cytokeratin (AE1/AE3) negativity excluded epithelial malignancy, but vimentin positivity supported a sarcoma lineage. Staining for myoglobin, a marker for rhabdomyosarcoma, was equivocal, and desmin was negative. A definitive diagnosis of rhabdomyosarcoma could not be made.

Our patient had a history of facial basal cell carcinoma, and metastatic lung tumors needed to be ruled out. However, as surgery and diagnosis were performed at another hospital, detailed pathological images were unavailable. Basal cell carcinoma is an epithelial tumor, which is inconsistent with the histological findings in this patient, who was negative for cytokeratin (AE1/AE3). Furthermore, metastasis of basal cell carcinoma is rare, reportedly occurring in 0.0001% of cases [14]. Based on these findings, the patient was diagnosed with malignant transformation from a mature cystic teratoma.

Non-seminomatous germ cell tumors are classified into four types, although there are no significant differences in treatment strategies; cisplatin-based chemotherapy, BEP therapy, and cisplatin and etoposide (PE) therapy when bleomycin is contraindicated are widely used [15]. Therefore, we chose BEP therapy.

In this case, despite gross incomplete resection during surgery, complete remission was achieved with postoperative BEP therapy. This therapy is effective in some cases of malignant transformation of mature cystic teratomas, suggesting that multidisciplinary treatment can improve prognosis. Had a detailed histological diagnosis been possible, the reasons for the effectiveness of BEP therapy may have been elucidated. In this case, multiple immunohistochemistry studies were performed, but a definitive diagnosis could not be reached. More cases need to be accumulated, and diagnostic accuracy must be improved by developing new immunohistochemistry techniques that can classify tumors into detailed histological types.

## Conclusions

We encountered a rare resected case of a malignantly transformed mature cystic teratoma in which remission was achieved with multidisciplinary treatment. There is no established standard chemotherapy for mature cystic teratomas that have transformed into malignant tumors; however, BEP therapy was effective in this case.

The histological types of mature cystic teratomas that have transformed into malignant tumors are complex; therefore, individualized treatment is important. To guide future investigations, more cases need to be accumulated and analyzed.
